# Contrasting effects of selective lesions of nucleus accumbens core or shell on inhibitory control and amphetamine-induced impulsive behaviour

**DOI:** 10.1111/j.1460-9568.2008.06309.x

**Published:** 2008-07

**Authors:** E R Murphy, E S J Robinson, D E H Theobald, J W Dalley, T W Robbins

**Affiliations:** 1 Behavioural and Clinical Neuroscience Institute and Department of Experimental Psychology, University of CambridgeCambridge, UK; 2 Department of Physiology and Pharmacology, School of Medical Sciences, University of BristolUniversity Walk, Bristol, UK; 3 Department of Psychiatry, University of CambridgeCambridge, UK

**Keywords:** behavioural inhibition, impulsivity, nucleus accumbens core, nucleus accumbens shell, rat

## Abstract

The core and shell subregions of the nucleus accumbens receive differential projections from areas of the medial prefrontal cortex that have dissociable effects on impulsive and perseverative responding. The contributions of these subregions to simple instrumental behaviour, inhibitory control and behavioural flexibility were investigated using a ‘forced choice’ task, various parameter manipulations and an omission schedule version of the task. Post-training, selective core lesions were achieved with microinjections of quinolinic acid and shell lesions with ibotenic acid. After a series of behavioural task manipulations, rats were re-stabilized on the standard version of the task and challenged with increasing doses of *d-*amphetamine (vehicle, 0.5 or 1.0 mg/kg i.p. 30 min prior to test). Neither core- nor shell-lesioned rats exhibited persistent deficits in simple instrumental behaviour or challenges to behavioural flexibility or inhibitory control. Significant differences between lesion groups were unmasked by *d-*amphetamine challenge in the standard version of the forced task. Core lesions potentiated and shell lesions attenuated the dose-dependent effect of *d-*amphetamine on increasing anticipatory responses seen in sham rats. These data imply that the accumbens core and shell subregions do not play major roles in highly-trained task performance or in challenges to behavioural control, but may have opposed effects following *d-*amphetamine treatment. Specifically, they suggest the shell subregion to be necessary for dopaminergic activation driving amphetamine-induced impulsive behaviour and the core subregion for the normal control of this behaviour via conditioned influences.

## Introduction

The nucleus accumbens (NAc) has been described as a ‘limbic–motor interface’ (Mogenson *et al.*, 1980), receiving input from prefrontal cortex (PFC) regions and structures such as the hippocampus and amygdala. The NAc core and shell subregions receive relatively discrete afferents from the prelimbic and infralimbic subregions, respectively (Berendse *et al.*, 1992; Brog *et al.*, 1993; Groenewegen *et al.*, 1999; Zahm, 1999, 2000; Vertes, 2004; Voorn *et al.*, 2004). Previous work has implicated the infralimbic cortex in the control of anticipatory responding in the five**-**choice serial reaction time task (5CSRTT) and ‘one’- or ‘forced’**-**choice (FC) task (Chudasama *et al.*, 2003a; Murphy *et al.*, 2005). In contrast, the prelimbic cortex has been implicated in perseverative responding in the 5CSRTT and FC task, as well as the development and maintenance of instrumental action–outcome representations (Chudasama & Muir, 2001; Coutureau & Killcross, 2003; Murphy *et al.*, 2005). These dissociations served as the rationale for the selection of specific core or shell lesions to elucidate the neural circuitry of inhibitory control and behavioural flexibility required by the 5CSRTT/FC task and related manipulations.

The contributions of the NAc subregions have not been thoroughly characterized in the 5CSRTT/FC tasks. Dorsal medial striatal lesions produced attentional deficits similar to those seen after medial PFC lesions, and dorsolateral striatal lesions severely impaired task performance and reacquisition (Rogers *et al.*, 2001). Christakou *et al.* (2004) demonstrated increased perseverative and slightly increased anticipatory responding after bilateral core lesions, but noted these effects only after non-rewarded trials. 6-OHDA lesions of the NAc in 5CSRTT-trained rats transiently reduced premature responding and slowed latencies, but did not otherwise affect baseline task performance (Cole & Robbins, 1989).

Previous studies have shown dissociable effects of core and shell subregions in a number of behavioural paradigms (Maldonado-Irizarry *et al.*, 1995; Weiner *et al.*, 1996; Parkinson *et al.*, 1999; Corbit *et al.*, 2001; Cardinal *et al.*, 2002; Ito *et al.*, 2004; Pothuizen *et al.*, 2005a, b, 2006). One objective of the present experiments was to analyse the behavioural flexibility required for adapting to changing task conditions and instrumental contingencies. The present investigation explored whether the NAc subregions play specific roles in modulating highly trained behaviour under changing task conditions and contingencies.

In the 5CSRTT, the effect of systemic *d-*amphetamine to increase premature (‘impulsive’) responding (Cole & Robbins, 1987, 1989; Harrison *et al.*, 1997; van Gaalen *et al.*, 2006; Pattij *et al.*, 2007) depends on dopamine-dependent mechanisms to the NAc (Cole & Robbins, 1987, 1989). However, given the interactive nature of these subregions in the control of behaviour (Weiner *et al.*, 1996; Parkinson *et al.*, 1999; Di Ciano & Everitt, 2001), we sought to determine their relative contributions to amphetamine-induced impulsivity. Based on interactions of core and shell lesions modulating psychostimulant properties and enhancing properties of *d-*amphetamine and cocaine on responding for conditioned reinforcer (Parkinson *et al.*, 1999; Ito *et al.*, 2004), it was hypothesized that lesions may have dissociable effects on premature responding elicited by *d-*amphetamine.

## Materials and methods

### Subjects

A single cohort of 36 male Lister Hooded rats (Charles River, UK), weighing 300–350 g at the start of the experiment, were used in this study. During behavioural testing, animals were maintained on 18 g of rat chow per day and had *ad libitum* access to water. Animals were housed in pairs under a reverse light cycle (lights on from 19:00 to 07:00 h) and testing took place between 09:00 and 19:00 h, 5–7 days/week. All experiments were carried out in strict accordance with the UK Animals (Scientific Procedures) Act 1986 under project license 80/1767.

### Behavioural procedures

Prior to surgery, animals were trained on the FC task as described below, modelled after the 5CSRTT (Carli *et al.*, 1983) and described by Dalley *et al.* (2002a). The task was implemented in the fivechoice program (Cardinal, 2002) using the Whisker control system (Cardinal & Aitken, 2001) running on PC computers (Dell, USA). Twelve 25 × 25 × 25 cm nine-hole operant conditioning chambers (Campden Instruments, UK) were used, each contained within a ventilated and sound*-*attenuated chamber and illuminated by a 3-W house light. Nine evenly spaced square holes (2.5 × 2.5 × 4 cm) each containing a 3-W light were set into the curved aluminium wall at the rear of the box, 2 cm above the wire-grid floor. In the FC task, only the single centre hole was uncovered and available. An infrared beam located at the entrance to each hole enabled detection of nosepoke responses. A food magazine into which food pellets could be dispensed (Noyes dustless pellets, 45 mg; Sandown Scientific, UK) was located in the middle of the opposite wall. The distance between the centre hole at the rear of the box and the magazine was 25 cm. An infrared beam located horizontally across the entrance to the magazine allowed recording of entries.

Animals received 5–7 sessions per week until a high level of stable performance was reached (> 90 completed trials, ≤ 15% omissions). Each session consisted of 100 completed trials and lasted a maximum of 30 min. Rats were trained to make nosepoke responses into the aperture in the front array upon brief stimulus illumination of the light located therein. The duration of the stimulus light was gradually reduced over 12 stages from 30 to 0.5 s. To begin a trial, rats were required to insert their nose in the magazine to activate the infrared beam. The extinguishing of the magazine light signalled initiation of a trial, and the next stimulus was illuminated after an intertrial interval (ITI). Following stimulus presentation, a correct nosepoke response was rewarded with a food pellet and illumination of the magazine light. Retrieval of the reward by entering the magazine initiated the next trial. A response prior to stimulus onset (premature) or failure to respond in a limited hold period after stimuli presentation (omission) were punished by a 5-s timeout period during which the house light was extinguished and no reward was delivered. Subsequent responses in any hole after the initial correct response or within the timeout period following an omitted response were classified as perseverative responding and punished with a 5 s timeout period.

### Surgery

Animals were anaesthetized and secured in a stereotaxic frame (David Kopf Instruments, Tujunga, CA, USA), fitted with atraumatic earbars, with the incisor bar set at −3.3 mm relative to the interaural line for a flat skull position. Animals were anaesthetized with inhaled isoflurane, carried in medical oxygen, induced at 5% and maintained at 1–2% concentrations at a flow rate of 2 L/min. Anaesthetic gases were delivered through a nosecone fitted on the incisor bar of the stereotaxic frame (David Kopf Instruments). The skull was exposed and a dental drill used to make small holes in the skull above the site of the microinjection. Co-ordinates for all surgery were derived using a stereotaxic atlas (Paxinos & Watson, 1998) using bregma as the origin (Table 1). Postoperative local anaesthetic was applied to the surgical wound to aid in analgesia.

**Table 1:** Injection parameters for selective lesions of accumbens subregions

	Coordinates (mm)			Injection		
			
	AP	L	DV (SS)	Vol. (μL)	Time (min:s)	Diffusion (min:s)
Core (0.09 m quinolinic acid)	+1.2	±1.8	−7.1	0.3	3:00	3:00
Shell (0.06 m ibotenic acid)	+1.6	±1.1	−7.9	0.16	1:30	1:30
			−6.9	0.1	1:00	1:00
			−6.4	0.1	1:00	1:00

AP, anteroposterior; L, lateral from midline; DV (SS), dorsoventral; SS, skull surface.

Different excitotoxins were used to selectively lesion the nucleus accumbens core or shell, using procedures similar to those in Parkinson *et al.* (1999) and Ito *et al.* (2004), known to produce selective lesions of each structure with little if any overlap between the damaged regions. A 1-μL SGE syringe was lowered directly into the core or shell structures and the toxin infused bilaterally. For core lesions, 0.09 m quinolinic acid (Sigma-Aldrich) buffered to pH 7.3–7.4 in 0.1 m phosphate-buffered saline (PBS) was injected according to parameters in Table 1. For shell lesions, 0.06 m ibotenic acid (Sigma-Aldrich, UK), buffered to pH 7.3 in 0.1 m PBS was injected according to parameters in Table 1. Lesion groups were counterbalanced for performance prior to surgery. Twelve rats were given each lesion, and six more ‘sham’ rats for each lesion area were treated identically except that PBS was infused instead of excitotoxin.

### Behavioural testing

**Fig. 1:**
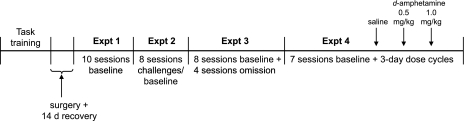
Schematic timeline of behavioural experiments. (Proportions of time blocks not to scale.)

A timeline in Fig. 1 illustrates the sequence of behavioural experiments. After 14 days of recovery, during which rats were pair-housed and had free access to food and water, they were again placed on a restricted food schedule (18 g/rat/day) and behavioural testing resumed. All post-surgical tests were completed within 4 months of surgery.

#### Experiment 1

Animals were tested with baseline (standard) task parameters for 10 consecutive days.

#### Experiment 2: attentional and inhibitory control challenges

In the FC task, animals were presented with the following challenge sessions: (i) shortened stimulus duration (250 ms) (ii) two consecutive long-ITI (7 s) sessions, (iii) variable ITI session (3.0, 4.5, 6.0 and 7.5 s ITIs), and (iv) high event rate (2 s ITI), with intervening baseline days using standard parameters.

#### Experiment 3: omission task

Animals were re-stabilized on the standard FC task but without the perseverative response punishment contingency and with a 2 s limited hold period until stable performance was re-established (eight sessions). Rats were then tested on four consecutive sessions of a new task with the instrumental contingency changed to an omission schedule: a nosepoke response to the stimulus light, or within the 2 s limited hold period, cancelled the reward and triggered a 5 s timeout period. To successfully perform the task and earn rewards, the rat merely had to start a trial by pressing the rear magazine door when cued by the light, withhold responding to the stimulus light, and collect the reward delivered 7 s later. An instrumental yoked control group was used to control for frequency of food reward; in this control, the positive nosepoke contingency remained intact but the availability of reward on a given trial was determined by the outcome (successful = omission or failed = nosepoke response) of the same trial for the ‘master’ rat under the omission contingency. Thus, rats in the yoked condition experienced the same rate of reinforcement as the rats in the omission contingency and were in essence performing the FC task on a partial reinforcement schedule. Rats were assigned to omission or yoked conditions within their lesion groups and remained in those conditions for the four consecutive days of the omission schedule test, hence in the analysis there are two between-subjects factors, Lesion (core, shell, or sham) and Condition (master or yoked) and one within-subjects factor, Trialblock (blocks of 10 trials) with 40 levels. The primary variable of interest for the omission task is the number of nosepokes made into the front array hole, and this measure can be subdivided into premature, ‘perceived correct’ for yoked rats (the timing of the response was such that it occurred after the stimulus light and within the limited hold), and ‘perseverative’. Also of interest is the number of ‘successful omissions’ made in the omission contingency condition (this variable is irrelevant for the yoked condition with the positive nosepoke contingency still intact), and the number of perseverative food panel nosepokes, as an indication of transference of behaviour from the now-irrelevant front panel to the relevant rear panel. In the omission contingency, rats were still required to initiate trials by pressing the rear panel when indicated by the magazine light.

#### Experiment 4: *d*-amphetamine challenge

Following the omission test, rats were re-trained on the standard FC task for seven sessions until stable performance was firmly established. Rats were then treated with *d-*amphetamine (Sigma-Aldrich, UK) on a 3-day cycle (baseline, drug treatment, home cage rest day) and in an ascending dose–response curve (saline, 0.5 and 1.0 mg/kg). All doses were administered i.p. 30 min prior to test at 1 mL/kg volume.

### Assessment of lesions

After behavioural testing was complete, subjects were anaesthetised with a lethal dose of sodium pentobarbital (Euthatal, 200 mg/mL; Genus Express, UK) and perfused transcardially with 0.01 m PBS followed by 4% paraformaldehyde. The brains were removed and post-fixed in 4% paraformaldehyde at least overnight. Prior to being sectioned, brains were transferred to a 20% sucrose solution in 0.01 m PBS as a cryoprotectant and left overnight. For lesion analysis, coronal sections were cut at 40 μm and every third section was taken for immunocytochemistry for visualization of the neuron-specific marker NeuN using monoclonal antibodies (MAB377; Chemicon International, UK) and a standard Vectatstain avidin–biotin procedure. Lesions were verified by light microscope examination of areas and cell damage was noted by lack of neuronal staining. The extent of lesions were mapped onto standardised sections of the rat brain (Paxinos & Watson, 1998).

### Statistical analysis

Behavioural data (omissions, premature responses, perseverative nosepokes, perseverative panel pushes, correct response latency and reward collection latency) were subjected to anova using a general linear model, using spss’s (v. 12.0.1, Chicago, IL) Type II sum-of-squares method, with appropriate between-subjects factors (i.e. Lesion) and within-subjects factors for each experiment. Homogeneity of variance was verified using Levene's test (Levene, 1960) and skewed data were subjected to appropriate transformations. All tests of significance were performed at α = 0.05. For repeated-measures analysis, Mauchly's test of sphericity (Mauchly, 1940) of the covariance matrix was applied and the degrees of freedom (df) were corrected to more conservative values using the Huynh–Feldt epsilon ɛ (Huynh & Feldt, 1970) to correct any violations in the sphericity assumption (Cardinal & Aitken, 2006). Corrected df are reported. Significant main effects of interest were investigated further using pairwise comparisons, with a Sidak correction for within-subjects factors with > 3 levels. If the significant main effect pertained to a factor with only three levels (as in Lesion: core, shell, and sham groups; or Dose: saline, 0.5 and 1.0 mg/kg), uncorrected *t*-tests (Fisher's Least Significant Difference procedure) were used (Cardinal & Aitken, 2006). Significant results for between-group or interaction terms are reported.

## Results

### Histology

**Fig. 2:**
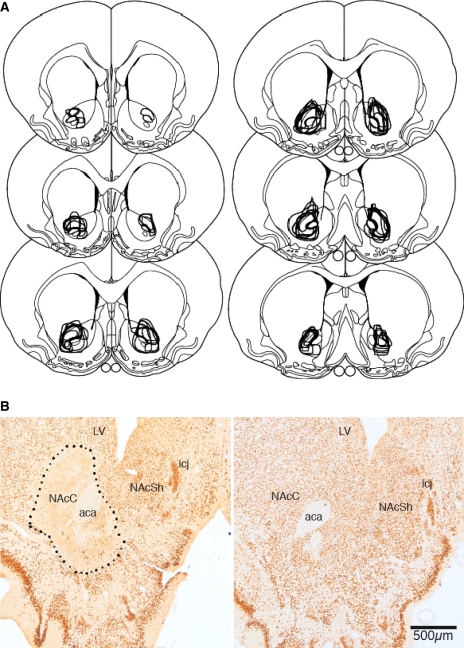
(A) Schematic diagrams of core lesions. From top left and going down columns, sections are +1.7, +1.6, +1.2, +1.0, +0.7 and +0.48 mm forward of bregma (Paxinos & Watson, 1998). Dark black lines outline the extent of each individual lesion in rats included in the analysis. (B) Photomicrographs of NeuN-stained coronal sections from core-lesioned (left) and sham-lesioned (right) animals. The lesioned area is indicated by the dotted line. Landmarks: *aca*, anterior commissure; LV, lateral ventricle; NAcSh, shell; NAcC, core; icj, islands of Cajella.

**Fig. 3:**
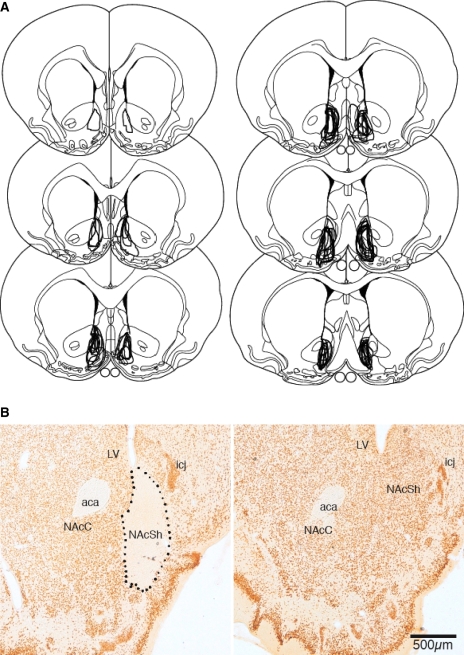
(A) Schematic diagrams of shell lesions. From top left and going down columns, sections are +1.7, +1.6, +1.2, +1.0, +0.7, and +0.48 mm forward of bregma. Dark black lines outline the extent of each individual lesion in rats included in the analysis. (B) Photomicrographs of NeuN-stained coronal sections from shell-lesioned (left) and sham-lesioned (right) animals. The lesioned area is indicated by the dotted line. Landmarks: aca, anterior commissure; LV, lateral ventricle; NAcSh, shell; NAcC, core; icj, islands of Cajella.

Eight of 12 core-lesioned rats were determined to have appropriate lesions, restricted to the core area of the NAc and not overlapping with the shell region (Fig. 2AandB). Nine of 12 shell-lesioned rats were determined to have appropriate lesions, restricted to the shell subregion and not overlapping with any other area (Fig. 3Aand B), with the lesion focused on the caudal shell. All 12 sham-lesioned rats showed no signs of cell damage aside from injector tracks, and were pooled for analysis as there were no behavioural differences between the sham rats receiving PBS in the core or shell regions. Final numbers (*n*) are provided in the results for each experiment.

### Behavioural results

#### Experiment 1: effect of core and shell lesions on baseline FC task performance (n = 8 core, n = 9 shell, n = 12 sham)

Neither lesion had significantly long-lasting effects on FC task performance. Core-lesioned animals made significantly more omissions as a proportion of total responses than sham- or shell-lesioned animals, but only on postoperative days 1–4 (Day × Lesion: *F*_5.4,70.5_ = 3.5, ɛ = 0.301, *P* = 0.008, followed by *post hoc* comparisions *P* < 0.05). Additional results are available in the Supplementary material.

#### Experiment 2: effect of core and shell lesions on attentional and inhibitory control challenges (n = 8 core, n = 9 shell, n = 12 sham)

A summary of these results is reported in Table 2.

**Table 2:** Summary of results for Experiment 2: attentional and inhibitory control challenges in FC task

	Short stimulus (0.25 s)	Short ITI (2 s)	Two consecutive long (7 s) ITI sessions	Variable ITI (3, 4.5, 6 or 7.5 s)
Omissions	–	–	–	–
Premature responses	–	–	All animals made fewer premature responses on Day 2	–
Correct response latency	Shell lesions faster than sham and core	Shell lesions faster than sham and core	–	Shell lesions faster than sham and core
Reward collection latency	–	–	–	–
Perseverative nosepokes	–	–	–	–
Perseverative panel pushes	–	–	All animals made fewer perseverative pushes on Day 2	–

Symbol ‘–’ indicates no differences between lesion groups.

#### Attentional challenges: shortened stimulus duration (250 ms) and increased event rate (short ITI) sessions

Significant between-groups differences were limited to shell-lesioned animals making faster correct responses than core-lesioned (*P* = 0.015) or sham-lesioned (*P* = 0.013) groups across all test conditions (shortened stimulus duration and shortened ITI; main effect of Lesion (*F*_2,26_ = 4.608, *P* = 0.019). No other outcomes of attentional challenges within the task were affected by different lesion groups.

#### Inhibitory control challenges: two consecutive long ITI sessions

Significant effects were limited to a main effect of Day on premature responses (*F*_1,26_ = 40.57, *P* < 0.001), where all animals made significantly fewer anticipatory responses on the second day of the long ITI session, but this result was not affected by lesion. A similar pattern of adaptation was seen for perseverative magazine entries (main effect of Day: *F*_1,26_ = 6.502, *P* = 0.017), with all animals making fewer perseverative entries on the second day of the long ITI, but no differences between lesion groups.

#### Temporal unpredictability: variable ITI session

The only significant effect of any lesion group was that, as for the attentional challenge sessions, shell-lesioned animals were faster than either core (*P* = 0.047) or sham (*P* = 0.02) to make correct nose-poke responses across all ITI lengths (main effect of Lesion: *F*_2,26_ = 5.091, *P* = 0.014).

### Experiment 3: effects of core and shell lesions on acquisition of omission contingency [n(core/master) = 4, n(core/yoked) = 4, n(shell/master) = 6, n(shell/yoked) = 3, n(sham/master) = 7, n(sham/yoked) = 4]

**Fig. 4:**
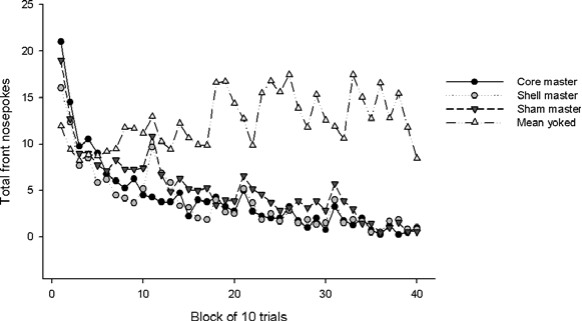
Neither core nor shell lesions affected the acquisition of the omission contingency. Yoked animals, by contrast, maintained front nosepoke responding under the intact instrumental contingency and a partial reward schedule. Graph illustrates total front nosepokes within blocks of 10 trials, over four consecutive sessions of 100 trials each. Yoked animals from each lesion group are averaged for clarity.

There were significant effects of omission contingency condition compared to yoked controls experiencing the same reward schedule, but neither core nor shell lesions affected acquisition of the omission contingency. Figure 4 compares total front nosepoke responses for animals experiencing omission contingency and the mean for yoked animals across lesion groups (no significant differences were found so groups were pooled for clarity). anova revealed a main effect of Trialblock (*F*_12.7,242.4_ = 4.302, ɛ = 0.326, *P* < 0.001) and a main effect of Condition (*F*_1,16 541_ = 32.7, *P* < 0.001), but no effect of Lesion and no Lesion × Condition interaction. Pairwise comparisons of interest show that, within the master (omission contingency) condition, there were no significant differences between lesion groups in the decline in the rate of nosepoking at the front apertures. In the instrumental yoked control condition, responding was sustained despite unpredictable frequency of reward as long as the instrumental contingency was intact and at least a partial reinforcement schedule was in effect.

The lack of lesion effects on acquiring the omission contingency was also analysed by ‘successful omissions’: main effect of Trialblock (*F*_31.5,56.7_ = 23.1, ɛ = 0.809, *P* < 0.001) but no effect of Lesion and no interaction. The core or shell lesions had no effect on the rate of acquisition of the omission contingency, which was achieved within four 100-trial sessions. In early trial blocks, rats were successfully omitting an average of three out of 10 trials. In late trial blocks, rats were successfully omitting an average of nine out of 10 trials.

### Experiment 4: effect of core or shell lesions on systemic d-amphetamine challenge (n = 8 core, n = 8 shell, n = 9 sham)

**Fig. 5:**
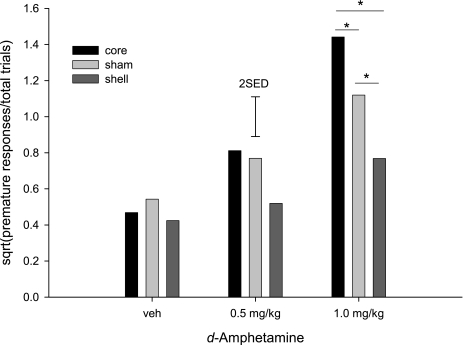
Shell lesions attenuated and core lesions exaggerated the effect of systemic amphetamine on the proportion of premature responses relative to sham animals. Graph illustrates transformed data for premature responses. SED (standard error of the difference) is for the pairwise comparisons of lesions vs. sham rats within the 1.0 mg/kg dose level. *P < 0.05 for pairwise comparisons within the 1.0 mg/kg dose level.

Administration of systemic amphetamine prior to task performance unmasked significant behavioural differences between lesion groups with respect to anticipatory responding. Raw data for premature responses violated homogeneity of variance assumptions (Levene's test), so data were transformed by taking the square root of each datum (Y′ = √(Y)), because variance was found to be proportional to the mean, and the arcsin transformation was unacceptable because several values of proportions of premature responses were > 1. (Transformed data are presented in Fig. 5 and untransformed means are available in the Supplementary material; the effect may be observed to be qualitatively the same.) anova revealed a main effect of Dose (*F*_2,44_ = 74.6, *P* < 0.001), a main effect of Lesion (*F*_2,22_ = 10.6, *P* = 0.001), and a significant Dose × Lesion interaction (*F*_4,44_ = 6.05, *P* = 0.001). Pairwise comparisons revealed a robust set of contrasting lesion effects. In sham-lesioned rats, 0.5 and 1.0 mg/kg doses caused a significant increase in premature responding relative to saline dosing (*P* = 0.011 and *P* < 0.001, respectively), and 1.0 mg/kg caused a further increase over 0.5 mg/kg (*P* = 0.002). The same pattern of significant dose–response effects was seen in core-lesioned rats and, furthermore, the effect of 1.0 mg/kg *d-*amphetamine on increasing premature responses was potentiated relative to the effect in shams (*P* = 0.008). In contrast, shell lesions markedly attenuated the effect of *d-*amphetamine on increasing premature responses. The 0.5-mg/kg dose of *d-*amphetamine had no effect on premature responses in shell-lesioned rats compared to saline (*P* = 0.284), and 1.0 mg/kg did increase premature responding relative to saline (*P* = 0.001) and 0.5 mg/kg (*P* = 0.024), but at the 1.0 mg/kg dose shell-lesioned rats made significantly fewer premature responses than core-lesioned rats (*P* = 0.001) and significantly fewer responses than sham-lesioned rats (*P* = 0.012). Thus core lesions potentiated, and shell lesions attenuated, increases in premature responding after 1.0 mg/kg amphetamine in intact rats.

Increasing doses of *d-*amphetamine increased the proportion of omitted responses (Dose: *F*_1.2,27.1_ = 5.82, ɛ = 0.616, *P* = 0.018). Increasing doses of *d-*amphetamine also increased perseverative responding (Dose: *F*_2,44_ = 9.51, *P* < 0.001), with a trend towards a Dose × Lesion interaction (core-lesioned rats made more perseverative responses after the highest dose of amphetamine) but this interaction did not reach significance (*F*_4,44_ = 2.3, *P* = 0.074). Shell-lesioned rats made significantly more perseverative panel pushes than core-lesioned rats (Lesion: *F*_2,22_ = 3.5, *P* = 0.049; *P* = 0.018) but neither core- nor shell-lesioned rats were different from shams. There were no significant effects of amphetamine or lesion on correct response latency or reward collection latency.

## Discussion

Selective lesions of the NAc core or shell, after training on a simple forced*-*choice instrumental task, had few major effects on performance under standard and challenge conditions. Neither lesion group showed a profile of distinctive, sustained impairments in task completion, inhibitory control, response latencies, behavioural flexibility or perseverative-type behaviour. Shell lesions did speed response latencies in behavioural challenge sessions, relative to core-lesioned or sham animals. This contrasts with previous findings that shell lesions produce locomotor hypoactivity (Parkinson *et al.*, 1999), and indicates that shell lesions do not invariably lead to reduction in motor output. One possible interpretation is that the shell lesion unmasks activating effects of novel test situations on the core subregion. All groups acquired a novel omission contingency at the same rate, suggesting no impairments in instrumental flexibility and, because of the omission nature of the contingency, no persistent impairments in response inhibition or perseveration. The major effects of interest were those elicited by systemic *d-*amphetamine administration. *d-*Amphetamine dose-dependently increased premature responding in sham rats. This effect was potentiated by lesions of the accumbens core and attenuated by lesions of the accumbens shell, without concurrent effects on response or reward collection latencies. These results suggest functionally opposed or co-modulatory roles of these accumbens subregions in the control of impulsive responding under certain conditions.

### Possible functional adaptation after core and shell lesions

Histological analysis of lesions confirmed that the use of different excitotoxins to target the core and shell (e.g. Parkinson *et al.*, 1999; Ito *et al.*, 2004) selectively produced circumscribed, discrete, and cell-body-specific lesions (Figs 2 and 3). Core and shell boundaries were determined based on visual inspection of relative density of NeuN immunoreactivity staining and position relative to anatomical landmarks (anterior commisure, islands of Calleja and lateral ventricles). While it is difficult to quantitatively determine the full extent and degree of completeness of a lesion in any individual subject, the qualitative group-level behavioural effects unmasked by *d-*amphetamine indicate that the lesions were sufficient to produce significant behavioural change and also selective in producing contrasting effects. The behavioural results over the immediate postoperative sessions suggest that functional compensation occurred and this may have masked subsequent lesion effects on performance of the behavioural challenge tests. This is consistent with the disruptive effect of acute infusions of the GABA agonist muscimol in the rat nucleus accumbens on the 5CSRTT (Murphy, 2007). However, the robustness of the damage caused by the lesion was demonstrated over 60 days post-surgery by the differential modulation of amphetamine effects. These results thus highlight important differences between cell-body lesions and ‘reversible lesions’ as produced by GABA agonist infusions. There is evidence for direct reciprocal connections between the shell and the core (van Dongen *et al.*, 2005) which, following a selective lesion of one subregion, may enable functional compensation for the other (Schoenfeld & Hamilton, 1977). The mechanisms of functional compensation may be facilitated by the highly trained and potentially habitual nature of the FC task (see below).

### NAc core and shell not implicated in behavioural flexibility

Behavioural outcomes for each task challenge were in line with previous reports of behavioural responses to task manipulations (Chudasama *et al.*, 2003a). Furthermore, the results from novel behavioural challenges (particularly the two consecutive long ITI sessions and the shift to an omission contingency) all imply that cell body lesions of the core or shell did not have major effects on behavioural flexibility. All animals showed increased premature responding when the ITI was initially lengthened from 5 to 7 s but on the second day of a 7 s ITI all groups of animals made fewer premature responses than the day before, an indication of intact behavioural adaptation. This result is consistent with the finding that core- or shell-lesioned animals were also unimpaired in acquisition of the omission contingency (Dickinson *et al.*, 1998), indicating intact behavioural flexibility.

The implication from the present work that the NAc core and shell are not involved in mediating different types of behavioural flexibility is seemingly inconsistent with recent work by Floresco *et al.* (2006) using a strategy-shifting task and by Pothuizen *et al.* (2005a) with a latent inhibition task. The discrepancy with the findings of Floresco *et al.* (2006) might be attributable to the chronic vs. acute nature of cell body lesions vs. GABA receptor agonist infusions (Lomber, 1999; and see above). However, the different requirements of the behavioural tasks may also contribute to the divergent results. Furthermore, such improvements in behavioural flexibility [such as the abolition of latent inhibition and hence facilitation of new learning (Pothuizen *et al.*, 2005a) and improving strategy shift (Floresco *et al.*, 2006)] were observed when lesions or inactivations were made prior to behavioural training and subsequent task performance depended in part on the ignoring of previously exposed stimuli or behavioural patterns. In the present study, demands on behavioural flexibility did not include any ‘irrelevant’ stimuli that would provoke response competition. In our experiment, shell lesions did not facilitate acquisition of the omission task, in which the previously learned action–outcome representation requires inhibition for the expression of the appropriate behavioural response to this contingency. Lesions performed prior to any behavioural training may have had significant effects on task acquisition; however, in the present experiment we were most interested in investigating post-training effects for comparison with the bulk of the 5CSRTT/FC task literature.

### NAc core and shell not implicated in normal or behavioural challenge-elicited impulsive responding

Baseline and behavioural challenge-elicited levels of premature responding were also not affected by NAc core or shell lesions. This lack of effect was initially surprising, in the light of the effects of core lesions reported by Christakou *et al.* (2004) to enhance impulsive responding after failed trials (a finding not replicated in the current study) and other work implicating the accumbens (particularly the core) in impulsive choice (Cardinal *et al.*, 2001). On the other hand, the present results are consistent with the finding that core lesions have no effect in mediating response inhibition in the stop-signal task (Eagle & Robbins, 2003). However, these tasks all measure different types of impulsivity (Evenden, 1999; Winstanley *et al.*, 2004a, 2006). Perhaps the closest behavioural parallel to the present task is the differential reinforcement of low rate (DRL) procedure. The FC task, lacking any component of spatial unpredictability, may be conceived of as a signalled DRL-5 task. Pothuizen *et al.* (2005a) found that core lesions impaired DRL performance, but this effect was not apparent on a DRL-4 schedule and did not emerge until DRL-12 and longer intervals (and the end of the interval was not signalled). Indeed, other work implicates the core in bridging delays longer than those experienced in the present task (Yun *et al.*, 2004; Cardinal & Cheung, 2005). Together, these results suggest that the NAc core may not mediate behavioural inhibition until a certain temporal threshold is exceeded, at which point it may function as a gateway between maintaining response preparedness and inhibiting motor output. Overall, the lack of effect of NAc lesions on most aspects of FC/5CSRTT performance is remarkable in comparison with the profound effects of dorsal striatal and subthalamic nucleus lesions (Baunez & Robbins, 1997; Rogers *et al.*, 2001; Chudasama *et al.*, 2003b), possibly reflecting the highly habitual nature of the task that would be consistent with the hypothesized roles of these brain areas in habit formation and maintenance (Packard & Knowlton, 2002; Grillner *et al.*, 2005; Yin & Knowlton, 2006).

### Shell lesions attenuated, but core lesions potentiated, effects of d-amphetamine on impulsive-type responding: convergent mechanisms

The most striking finding of the present experiment was that, despite no apparent effects of core or shell lesions on task performance, a lesion-specific divergence emerged in the behavioural response to systemic *d-*amphetamine with respect to premature responding. This result raises several interesting questions: the underlying mechanism of amphetamine-induced premature responding, the separate roles of the core and shell in mediating these effects, and how these roles converge with associative learning elements to allow the inhibition or execution of appropriate motor programs.

*d-*Amphetamine reliably increased premature responding in the FC task, replicating earlier findings in the 5CSRTT (Cole & Robbins, 1987, 1989; Harrison *et al.*, 1997; van Gaalen *et al.*, 2006; Pattij *et al.*, 2007). *d-*Amphetamine also has locomotor activating effects (which are also similarly modulated by core or shell lesions, as is the impulsive measure in the present result; Parkinson *et al.*, 1999). It is of particular interest that amphetamine had no effects on response or reward collection latencies, measures used in the 5CSRTT/FC task as an index of locomotor activation (see Table 2), indicating that amphetamine's activating effects probably operate on response selection rather than simply response speed *per se*. Indeed, shell lesions sped response latencies in challenging task conditions (see above) yet attenuated amphetamine-induced premature responding. Thus, increases in premature responses are not simply attributable to locomotor activation. Other findings also show that latency and premature response measures dissociate after various experimental manipulations: after rearing in social isolation (Dalley *et al.*, 2002b; Liu, 2002), which led to locomotor hyperactivity but reduced premature responses in the 5CSRTT, and in highly impulsive rats in the 5CSRTT which tend to be less active in novel photocell cages (Dalley *et al.*, 2007).

*d-*Amphetamine-induced premature responding clearly depends on the dopaminergic innervation of the NAc (Cole & Robbins, 1989). However, the data indicate that this dopamine-dependent effect in the NAc may particularly implicate the shell subregion. Previous data have indicated that the rate-increasing effects of *d-*amphetamine and cocaine on operant responding probably similarly depend primarily on the shell subregion (e.g. Parkinson *et al.*, 1999; Ito *et al.*, 2004). The present dissociation between effects of core and shell lesions implies competing effects of amphetamine acting in different subregions of the NAc, which have also previously been characterized as having competing functional influences in the latent inhibition paradigm (Weiner, 2003). Amphetamine exerts different effects across core and shell territories, and may act preferentially in the shell (Heidbreder & Feldon, 1998; Dalley *et al.*, 1999). Given the reciprocal interconnection between the core and the shell (van Dongen *et al.*, 2005), lesions of one subregion might thus eliminate modulatory inputs, allowing amphetamine to act in core or shell exclusively and hence exaggerating its effects.

The NAc shell has been reported to mediate the degree to which Pavlovian conditioned factors affect instrumental responding. For instance, shell lesions abolished Pavlovian-to-instrumental transfer, a potentiation of instrumental responding seen when a conditioned stimulus previously paired with reward delivery is introduced into an instrumental condition in which the animal is responding for the same reward (Corbit *et al.*, 2001). Shell lesions also decreased the potentiation of responding for conditioned reinforcement elicited by amphetamine (Parkinson *et al.*, 1999). Intra-shell amphetamine potentiated Pavlovian cue-elicited instrumental performance (Wyvell & Berridge, 2000). In the present results, shell lesions significantly attenuated (without completely blocking) the extent to which 1.0 mg/kg of amphetamine increased premature responses. This effect may be akin to blocking the rate-increasing effects of behavioural arousal elicited by Pavlovian conditioned contextual stimuli that may normally be processed via hippocampal afferents to the shell subregion (Groenewegen *et al.*, 1996, 1999).

By contrast, the effects of the conditioned stimulus (in this case, the stimulus light target in the 5CSRTT/FC task) may be mediated by the core. The core has previously been shown to mediate the conditioned reinforcing effects of such stimuli (Parkinson *et al.*, 1999; Ito *et al.*, 2004). However, in this situation, the target light, as a result of its association with food reward, may exert inhibitory control over premature responding; i.e. in its absence, nose-poke responding is suppressed. The core lesion would therefore lead to an inappropriate increase in premature responding, thus ‘channelling’ the behavioural activation produced by amphetamine's effect in the shell into the release of behavioural inhibition. How precisely the shell-dependent effect of the drug normally interacts with core-dependent mechanisms is unclear, although it is probably related to the ‘cascading shell-to-core’ loop circuitry described by Haber *et al.* (2000). Thus inputs to the shell may influence the core, which eventually influences dorsolateral striatal structures, channelling information via ‘cascading’ reciprocal and non-reciprocal feedback and feedforward loops to impact on final motor outcome.

## Conclusions

The present work has demonstrated that the NAc core and shell have only limited roles in mediating performance of a basic serial reaction task and in the modification of responding to attentional demands, changes in contingency and inhibitory control. However, there were contrasting effects of core lesions (exacerbating) or shell lesions (attenuating) the potentiation of premature responding in the FC task by systemic amphetamine, suggesting some co-modulatory functions of these accumbens subregions in the expression of certain forms of impulsive behaviour.

## Abbreviations

5CSRTT: five-choice serial reaction time task
DRL: differential reinforcement of low rate
FC task: forced-choice task
ITI: intertrial interval
NAc: nucleus accumbens
PBS: phosphate-buffered saline
PFC: prefrontal cortex
